# The Experience of a Country Practice Necessary for a Complete Medical Training

**Published:** 1917-02

**Authors:** C. T. Bradford

**Affiliations:** Klondike, Texas


### The Experience of a Country Practice Necessary for a Complete Medical Training.

BY DR. C. T. BRADFORD, KLONDIKE, TEXAS.

The progress of time, as far back as we have knowledge, has not been without the physician, although he has played many a rôle on the stage of human action. In the beginning of his career, so far as history gives us information, the physician, or healer as he was called, was a person supposed to have the power to cast out evil spirits, and thus we see him in the evolution of the profession from the age of superstition and idolatry to the present well-trained man of today.

Most of the States of our great Union have a high standard of medical education at present, and yet, realizing that life is too precious to be wasted at the hands of imposters, and the mortality still too high, this standard is being raised each decade, and we hope to some day see it reach a point where the medical man shall have attained the zenith of thoroughness. Even now many State boards are requiring a certain period of interneship before an applicant is eligible to take the examination.

Psychological medicine has had no place in our colleges, or even to any great extent in the practice until the last few years, and we now believe it to have a very important place, especially in the treatment of nervous conditions.

Very unfortunately the average physician is a man of limited means and must hasten to an active practice, after leaving school, in order to repay some good Samaritan who has helped him through, without getting the advantage of some years of hospital work, and is really unfit to merit the confidence which the people should place in the family doctor. Our paper, however, will presume that every man who announces himself as one of the profession has had the four years' course of study, together with the required interneship, and shall deal more with the contrast between those who practice in the city, with access to the hospitals and laboratories, and those who do a country practice, with access to nothing except their own resources, as we realize that an education means development to the highest degree of the mental faculties, which is done by continual activity.

No doubt all of you who began your medical career in the country have recollections of being called in the dead hours of the night, and on the journey to the sick room, having gathered all the information possible from the messenger, who came on horseback for you, in order that the diagnosis might be almost complete by the time you had reached the home, only to have your most favorable impression, which you had expected to make, shattered by an entirely different chain of symptoms than those pictured. Still, it was absolutely necessary that you should make the diagnosis before leaving the home, for the mother and father were already alarmed at the child's condition, and could not realize that the treatment would be one to meet the symptoms rather than the disease, as we know very few specifics.

If it is your good fortune to be located in a city, with a well equipped laboratory a few blocks away, it is usually easy to satisfy the family by meeting the immediate conditions by taking a specimen of blood, sputum, urine or feces, and making a complete diagnosis after receiving the facts as reported to you by the pathologist. This, of course, means that we become more and more accustomed to allowing the other man to really make the diagnosis for us.

The man in the country must make some preparation to do his own laboratory work, and thus each examination familiarizes him more with the real pathological pictures than he can hope to become if dependent upon a word picture drawn by the other man. Do not undertsand me to even intimate that the country practitioner is better prepared to practice his profession than his city brother, for, on the contrary, he is less able to meet the ideals prescribed by our text-books, on account of his environment.

Being without trained help and knowing that he must wait some hours before the neighboring physician can be gotten, with no light except the small amount given by one oil lamp, and the patient rapidly losing from hemorrhage into a cavity or in a state of eclampsia necessarily makes him inventive, and leads him to depend more upon his own knowledge than he would if a hospital, with its trained nurses, laboratories and sterile rooms, were at hand.

I imagine a man who has had no experience except that gotten inside the walls of a sanitarium would, for a time, be confused if, after answering a hurry call to some family he had never known of, he should find a case of obstetrics, in the second stage, and the surroundings indicating sepsis on every hand, even to the extent that the parturitrant had not received the preparatory bath, nor the linen been changed for a number of days, as she thought it would be unnecessary as all would have to be done after the confinement. Indeed, the man who could meet such state of affairs calmly and without some confusion, even though they were daily occurrences, must certainly not have his soul in the one thought of relieving the world of distress. Such examples as these may seem strange to some of you, yet, although such conditions are rarely met, they do exist, and it happens to be the lot of the country doctor to contrive some means of handling them to the best advantage for the patient. Just as perplexing problems as the one just mentioned confront us in such circumstances as caring for an epidemic of some contagious disease. The father, who is the only salaried person on the place, must, if possible, continue to go to and from his work in order that the mother and six children may be provided with the necessities of life, and yet there is no detention camp or isolated ward to take care of the case of smallpox. There is no nurse to be with it in order that the mother may watch the other children, but something must be done to solve the problem, and the faithful family doctor is the one who is called upon for this advice. in surgery, necessitated by accidents, for that is about the only Only a short time ago I was called at midnight to attend a boy who had a knife wound in the abdominal wall just over the appendix. The gash was about two inches long, and had penetrated to the cavity in a small place. The patient had been bleeding all the time while he drove in a buggy the distance of six miles, but his recovery was expected to be uneventful even though there was not so much as hot water at hand; and again in combating the advice distributed by the neighborhood gossip, who tells your patients to seal all cracks in the walls and never change clothing or give baths where there is a pneumonia patient. I have one time seen the patient die from a general septic condition contracted after a large surface had been blistered, and the patient had lain several days without having even the fecal matter removed from clothing and person.

I have often wondered after reading a chapter of some textbook, or hearing a manuscript, if the author would not have changed it somewhat had he come in contact with a few of these obstacles in active practice before writing it.

Knowing that, away off in some remote corner of civilization, there is a man who is trying to save lives under such adverse conditions, why should we feel that he should get the same results as those who are working in a field where asepsis and sanitation are paramount, and how do we figure that every case of puerperal septicemia or other infection is due directly to his negligence? Nevertheless, he is regarded as negligent and inefficient, and many times is forced to bear considerable burden because of the remark, "I am too late to be of assistance," made by a thoughtless consultant who doesn't realize fully the circumstances under which his brother is laboring.

Being versed then in the fundamental principles and technicalities of the profession, the experience gained through a country practice enables us to become more thorough in physical and clinical diagnosis, from having all this to do instead of sending it to the other fellow, better acquainted with the symptoms of the various maladies, because in the absence of a nurse we are forced to watch the patient longer when we call than would be necessary if an accurate chart were at hand to be studied hurriedly, and more devoted to the work because of the desire to ameliorate suffering which we are forced to witness so much more of than we would be under the more favorable surroundings.

Aside from the scientific part of medicine, the doctor should deserve, and must know, the art of securing the unqualified confidence of his patrons, and we feel that this can best be learned among the country folk who know nothing of the rush of city life, where too often the ministrations of the faithful family doctor are looked upon, not in the sympathetic manner in which they are given, but as selfish business transactions.

---

There has not been a single case of yellow fever in the United States since 1905.

